# Special Issue: Alzheimer's disease

**DOI:** 10.3934/Neuroscience.2018.1.74

**Published:** 2018-01-26

**Authors:** Khue Vu Nguyen

**Affiliations:** 1 Department of Medicine, Biochemical Genetics and Metabolism, The Mitochondrial and Metabolic Disease Center, School of Medicine, University of California, San Diego, Building CTF, Room C-103, 214 Dickinson Street, San Diego, CA 92103-8467, USA; 2 Department of Pediatrics, University of California, San Diego, School of Medicine, San Diego, La Jolla, CA 92093, USA

**Keywords:** Alzheimer's disease, Amyloid-β (Aβ) peptides, Neurofibrillary tangles (NFTs), β-amyloid precursor protein (APP), Familial AD (FAD), Sporadic AD (SAD), Epigenetic modifications, gene-gene and/or gene-environment interactions

## Abstract

More than 45 million people worldwide have Alzheimer's disease (AD), a deterioration of memory and other cognitive domains that leads to death within 3 to 9 years after diagnosis. The principal risk factor for AD is age. As the aging population increases, the prevalence will approach 131 million cases worldwide in 2050. AD is therefore a global problem creating a rapidly growing epidemic and becoming a major threat to healthcare in our societies. It has been more than 20 years since it was first proposed that the neurodegeneration in AD may be caused by deposition of amyloid-β (Aβ) peptides in plaques in brain tissue. According to the amyloid hypothesis, accumulation of Aβ peptides, resulting from a chronic imbalance between Aβ production and Aβ clearance in the brain, is the primary influence driving AD pathogenesis. Current available medications appear to be able to produce moderate symptomatic benefits but not to stop disease progression. The search for biomarkers as well as novel therapeutic approaches for AD has been a major focus of research. Recent findings, however, show that neuronal-injury biomarkers are independent of Aβ suggesting epigenetic modifications, gene-gene and/or gene-environment interactions in the disease etiology, and calling for reconsideration of the pathological cascade and assessment of alternative therapeutic strategies. In addition, recent research results regarding the expression of the β-amyloid precursor protein (*APP*) gene resulting in the presence of various APP-mRNA isoforms and their quantification, especially for identifying the most abundant one that may decisive for the normal status or disease risk, have been reported. As such, a more complete understanding of AD pathogenesis will likely require greater insights into the physiological function of the β-amyloid precursor protein (APP).

## 1. Introduction

It is with great pleasure to introduce a special issue, namely “Alzheimer's disease”, which is scheduled to appear this year in AIMS Neuroscience. I cordially invite authors to contribute their excellent works to this exciting forum. Submissions are now open and will be fully considered for publication.

Alzheimer's disease (AD) is renowned as the most prevalent multifactorial neurodegenerative disorder in today's world. It is the most common cause of dementia in the elderly, affecting about 46.8 million persons worldwide, and will reach a height of 74.7 million in 2030 and 131.5 million in 2050 [1]. In AD, a deterioration of memory and other cognitive domains that leads to death within 3 to 9 years after diagnosis. Owing to the dramatic increase in the population as the year and age progresses, AD is not only considered to be life threatening but also an economic and social burden to the health-care system. Since 1992, the amyloid cascade hypothesis has played a prominent role in explaining the etiology AD. It proposes that the deposition of amyloid-β (Aβ) peptides, resulting from a chronic imbalance between Aβ production and Aβ clearance, is the initial pathological event in AD leading to the formation of extracellular senile plaques (SPs) and then to intracellular neurofibrillary tangles (NFTs) of hyperphosphorylated tau proteins, neuronal cell death and, ultimately, dementia [2]. Aβ peptides are natural products of metabolism consisting of 36 to 43 amino acids. Monomers of Aβ_40_ are much more prevalent than the aggregation-prone and damaging Aβ_42_ species. Aβ peptides originate from proteolysis of the β-amyloid precursor protein (APP) by sequential enzymatic action of beta-site amyloid precursor protein-cleaving enzyme 1 (BACE-1), a β-secretase, and γ-secretase, a protein complex with presenilin 1 at its catalytic core [3]. AD is currently classified by age at onset and genetic status [4]. Sporadic AD (SAD) is characterized by late age at onset (onset age >65 years) and accounts for ∼99% of AD cases. Familial AD (FAD) accounting for ∼1% of cases, is characterized by early age at onset (onset age <65 years) and a genetic component [4]. In these cases, mutations in the *APP* gene, and the *presenilin* (*PS*) genes *PS1* and *PS2* are known to be associated with FAD.

## 2. Biomarkers

Advances in research have been translated into several drug candidates with disease-modifying potential, many of which are now being evaluated in clinical trials [5]. Studies in transgenic mouse models of AD suggest that the majority of these types of disease-modifying drugs may be most effective early on in the process of Aβ aggregation, and be less effective in later stages when there is severe plaque pathology and neurodegeneration [6]. However, the current diagnostic criteria from the National Institute of Neurological and Communicative Disorders and Stroke and the Alzheimer's Disease and Related Disorders Association (NINCDS-ADRDA) that are used to identify patients with AD who have overt dementia correspond to neuropathologically advanced disease. Consequently, early recognition of the disease needs to be improved. Indeed, it is estimated that interventions that could delay the clinical onset of dementia by 1 year would reduce the prevalence in 2050 by 9 million cases [6]. Several multimodal core biomarkers candidates derived from structural, functional and metabolic neuroimaging, and from neurochemistry and genetic studies of AD have been investigated but their values in pivotal efficacy studies for AD are still limited [6]. Examples of genetic analysis in biomarker research include testing for *PS1, PS2*, and *APP* mutations; however, as *PS1*, *PS2*, or *APP* mutations are rare, screening for these mutations currently has to be organized on an ad hoc basis. In addition, over the last decades, the apolipoprotein E gene (*APOE*) with three variants referred as *APOE* ε2, ε3 and ε4 alleles, has been irrefutably recognized as a major risk factor for SAD. Unlike *APP, PS1, and PS2* mutations that are fully penetrant (causal), *APOE* ε4 allele is the most important genetic risk factor for SAD. Only about 20–25% of the general population carries one or more ε4 alleles, whereas 50–65% of people with AD are ε4 carriers. The presence of at least one ε4 allele has ben associated with reduced age at onset rather than increases in the lifetime risk of developing AD. Homozygous ε4 allele carriers develop AD up to 10 years earlier than individuals who do not have this allele. Nonetheless, *APOE* ε4 does not account for all genetic variation in AD. The use of *APOE* as a diagnostic or predictive factor in clinical practice is therefore not warranted. Recent works have demonstrated a rare functional variant (R47H) in triggering receptor expressed on myeloid cells 2 gene (*TREM2*), encoding TREM2 transmembrane glycoprotein, increase susceptibility to SAD, with an odds ratio similar to that of the *APOE* ε4 allele [7],[8]. Emerging evidence has demonstrated that TREM2 could suppress inflammatory response by repression of microglia-mediated cytokine production and secretion, which may prevent inflammation-induced bystander damage of neurons. Proteolytic processing of TREM2 results in release of a soluble, ectodomain-containing TREM2 fragment (sTREM2) that is detectable in cerebrospinal fluid (CSF) and serum. Changes in the levels of AD-related proteins in CSF, such as Aβ_42_ and tau, reflect the presence and progression of AD pathology in the brain. Studies of SAD and autosomal-dominant AD populations found that sTREM2 levels were increased in the CSF of AD patients and that sTREM2 levels were positively correlated with levels of tau and phosphorylated tau (p-tau); however, no robust correlation between sTREM2 and Aβ_42_. This suggests that sTREM2 levels increase in association with neuronal damage resulting from an inflammatory process in AD rather than with the appearance of Aβ plaques per se. Then, much work remains to both validate the relationship between sTREM2 and AD and understand the mechanism underlying a potential relationship between TREM2 and neuronal toxicity. Although several studies indicate that elevated sTREM2 levels may mark the transition from preclinical AD to symptomatic AD, some studies have reported contrary findings of either decreased or unchanged CSF sTREM2 levels in AD patient populations. In addition, although most of studies support the hypothesis that inflammation is present as a pathogenetic force in the process of AD, it still remains unclear whether inflammation is a causative factor or just a consequence of this disease. Understanding the role of TREM2 in regulating the response of the innate immune system to Aβ and/or tau pathology may lead to the discovery of novel biomarkers and therapeutic strategies. Finally, up to present, none of the imaging, genetic or biochemical markers has been sufficiently qualified as a surrogate for the actual NINCDS-ADRDA. There is a growing need for biomarkers of AD pathology to improve drug development related to the disorder. Indeed, biomarkers are needed to monitor drug safety, to identify individuals who are most likely to respond to specific treatments, to stratify pre-symptomatic patients and to quantify the benefits of treatments [6].

## 3. Treatment strategies

Up to now, six broad therapeutic strategies based on Aβ biology have been proposed [5],[9]. First, one could attempt to partially inhibit either of the two proteases, β- and γ-secretase, that generate Aβ from APP. Second, one could attempt to prevent the oligomerization of Aβ or enhance its clearance from the cerebral cortex. The third broad approach is an anti-inflammatory strategy based on the observation that a cellular inflammatory response in the cerebral cortex is elicited by the progressive accumulation of Aβ. The fourth approach is based on modulating cholesterol homeostasis. The fifth strategy is based on the observation that Aβ aggregation is, in part, dependent on the metal ions Cu^2+^ and Zn^2+^. The sixth broad amyloid-based strategy is to prevent the synaptotoxic and neurodegenerative effects putatively triggered by Aβ accumulation. Numerous approaches have been contemplated, including the use of compounds with antioxidant, neuroprotective, and/or neurotrophic properties, but again, no slowing of cognitive decline has been documented in human to date. Thus, for now, clinically validated treatments for AD remain confined to symptomatic interventions such as treatment with acetylcholinesterase inhibitors such as donepezil, rivastigmine, galantamine, huperzine-A, and memantine (a partial antagonist of N-methyl-D-aspartate receptor, NMDAR), and drugs that ameliorate behavioral disturbances such as antidepressants, sleeping tablets, tranquilizers [5],[9].

## 4. Future directions

Although the amyloid cascade hypothesis has been dominated the field for more than 20 years, and offered a broad framework to explain AD pathogenesis, it is currently lacking in detail, and certain observations do not fit easily with the simplest version of the hypothesis [9],[10]. The most frequently voiced objection is that the number of amyloid deposits in the brain does not correlate well with the degree of cognitive impairment that the patient experienced in life. Furthermore, over recent years, data have illustrated that reciprocal interactions between APP and its various metabolites, including Aβ, can powerfully regulate key neuronal functions including cell excitability, synaptic transmission and neural plasticity [11]. As a consequence, perturbation of some of these activities may contribute to AD pathogenesis and neurodegeneration in an Aβ-dependent or Aβ-independent manner, and therefore (a) SPs and NFTs may be developed independently and able to interact with each other, and thereby promoting the AD neuropathological cascade, and (b) SPs and NFTs may be the products rather than causes of neurodegeneration in AD [11]. We are entering an era in which the unitary view of AD as a single sequential pathological pathway with Aβ considered as the only initial and causal event is likely to be progressively replaced by a more complex picture in which AD is considered as a multi-parameter pathology that is subtended by several partly independent pathology processes. In this disease, neuronal injury could be caused by different factors, with various possible sequences of pathological events. In contrast to monogenic disease, SAD exhibits numerous non-Mendelian anomalies that suggest epigenetic modifications, gene-gene and/or gene-environment interactions in the disease etiology. Compared to genetic causes, epigenetic factors are probably much more suited to explain the observed anomalies in SAD as aberrant epigenetic patterns may be acquired during many developmental stages. The epigenome is particularly susceptible to deregulation during early embryonal and neonatal development, puberty and especially old age, which is the most important known risk factor for AD [11]–[13]. Indeed, mutations in FAD represent a very small percentage (∼1%), and ∼99% of cases are SAD [4]. Multiple studies conducted to determine disease-causative loci have revealed that AD is highly complex and heterogeneous in nature. Therefore, non-genetic factors, such as epigenetic modifications, gene-gene and/or gene-environment interactions may also be causative and currently the subject of intense research [11]–[13]. As such, it is important to continue to investigate the normal function of APP. Understanding its physiological function will not only provide insights into the pathogenesis of AD but may also prove vital in the development of an effective therapy. Recently, reports on epigenetic regulation of alternative splicing of *APP* gene resulting in the presence of various APP-mRNA isoforms and their quantification, especially for identifying the most abundant one that may decisive for the normal status or disease risk, have been described [14]–[17]. These findings may provide new directions for the research in neurodevelopmental and neurodegenerative disorders in which the *APP* gene is involved in the pathogenesis of diseases such as autism [18],[19], fragile X syndrome [19], amyotrophic lateral sclerosis [20], multiple sclerosis [21], and AD [10],[11],[19], and may pave the way for new strategies applicable to rational antisense drugs design [22].

In conclusion, it is clear that APP undergoes complex regulation and is important for neuronal and synaptic function in both central and peripheral nervous systems. This may involve the APP extracellular domain, the APP intracellular domain, the Aβ sequence or, indeed, cross communication among these motifs [11]. Then, *APP*, a house keeping gene [23], and endogenous ligand (http://www.genenames.org/genefamilies/EnDOLIG), is an important molecular hub at the center of interacting pathways and acts as a permissive factor for various neurodevelopmental and neural circuit processes [24], altered APP processing may affect brain function through a host of altered cellular and molecular events occurring in neurodevelopmental and neurodegenerative disorders such as autism, fragile X syndrome, and AD. As such, a more complete understanding of AD pathogenesis will likely require greater insights into the physiological function of APP.
